# Throat related symptoms and voice: development of an instrument for self assessment of throat-problems

**DOI:** 10.1186/1472-6815-10-5

**Published:** 2010-05-27

**Authors:** Viveka Lyberg-Åhlander, Roland Rydell, Jacqueline Eriksson, Lucyna Schalén

**Affiliations:** 1 Department of Logopedics, Phoniatrics and Audiology, Lund University, S-221 85 Lund, Sweden; 2 ENT-Department, Lund University Hospital, S-221 85 Lund, Sweden

## Abstract

**Background:**

Symptoms from throat (sensation of globus; frequent throat clearing; irritated throat) are common in patients referred to voice clinics and to ENT specialists. The relation to symptoms of voice discomfort is unclear and in some cases patients do not have voice problems at all. Instruments for patients' self-reporting of symptoms, and assessment of handicap, such as the Voice Handicap Index (VHI), are in common use in voice clinics. Symptoms from throat are however only marginally covered. Purpose: To develop and evaluate an instrument that could make the patients' estimation of symptoms from the throat possible. Further to facilitate the consideration of the relation between throat- and voice problems with the Throat subscale together with a Swedish translation of the Voice Handicap Index. Finally to try the VHI with the Throat subscale: the VHI-T, for test-retest reliability and validity.

**Methods:**

A subscale with 10 throat related items was developed for appliance with the VHI. The VHI was translated to Swedish and retranslated to English. The questionnaire was tried in two phases on a total of 23+144 patients and 12+58 voice healthy controls. The reliability was calculated with Cronbach's alpha, ICC and Pearson's correlation coefficient. The validity was estimated by independent T-test.

**Results:**

The difference in VHI-T scores between the patients and the voice-healthy controls was significant (*p *= < 0,01) and there was a good correlation of the test- retest occasions. The reliability testing of the entire questionnaire showed an alpha value of *r *= 0,90 and that for the Throat subscale separately a value of *r *= 0,87 which shows a high degree of reliability.

**Conclusions:**

For the estimation of self-perceived throat and voice problems the scale on throat related problems together with the present Swedish translation of the Voice Handicap Index, (VHI) the VHI-Throat, proves to be a valid and reliable instrument. The throat subscale seems to help revealing a category of symptoms that are common in our patients. These are symptoms that have not earlier been possible to cover with the questionnaires designed for use in the voice clinic.

## Background

Patient-reported symptoms together with laryngostroboscopy and perceptual analysis of the voice are essential for the evaluation of voice in logopedic and phoniatric practice [1,2]. A number of instruments for the self-rating of voice problems have been developed for use in the voice clinic. The Voice Handicap Index (VHI) [3] along with the shortened VHI: VHI-10 [4]; the Voice Activity and Participation Profile (VAPP)[5]; the Voice-Related Quality of Life (VrQoL)[6]; the Voice Outcome Survey (VOS) [7] and the Voice symptom scale (VoiSS) [8] are all designed for measuring perceived handicap and quality of life, and perceived limitations of participation and activity.

Symptoms related to the throat, such as frequent throat clearing, irritated throat, sensation of globus, or foreign body are frequently reported by patients suffering from voice disorders. These symptoms are, however, not specific and maybe due to a multitude of underlying disorders. In the area of voice, throat symptoms may be interpreted either as the *cause *of functional voice disturbances and in reverse they may also be interpreted as a *consequence *of voice load or inappropriate vocal behavior [9]. Apart from vocal behavior, non-specific mucosal hyperreactivity [10], laryngo-pharyngeal reflux [11], allergy [12] and mass lesions in the throat region are often considered as causative factors. Thus, throat related problems are a rather common concern in patients referred to voice clinics. To our knowledge, among the afore-mentioned questionnaires designed for the self-evaluation of voice problems, only the VoiSS questionnaire includes a number of items addressing pharyngeal symptoms [8]. To have a complete overview of the voice-related problems, and to meet the needs of this group of patients, this type of symptoms should also be better understood. Three self-assessment scales address only the issue of throat related symptoms; however, all scales are designed to measure problems of more diagnose-specific character, the Glasgow and Edinburgh Throat Scale, designed for the evaluation of globus [13], the Reflux Symptom Index [14] and the Pharyngeal Reflux Symptom Questionnaire (PRSQ) [15], which specifically addresses reflux.

Our aim with this paper was to develop and evaluate an instrument that could simplify the patients' estimation of symptoms from the throat and to consider their relation to voice problems simultaneously. The Voice Handicap Index (VHI), a multidimensional, self administered questionnaire, developed and validated by Jacobson et al. in 1997 [3] has been translated into many languages and is widely used in clinical work and research, with at least 200 publications up till today. At our clinic, the VHI has been in use since 2000 along with a subscale designed for the measurement of throat related symptoms, VHI-T. The VHI is an instrument that is easy to distribute and to analyze. We considered it of importance for the patient to have the possibility to judge all perceived voice- and throat symptoms in the same manner and within the same "formula", by keeping to the same rating scale and number of statements as well as to the way of phrasing the statements. We therefore choose to follow the structure of the VHI, which consequently gives the possibility to use the throat-scale as a supplement to the original VHI. The present paper thus describes the construction and validation of a scale on throat symptoms in voice patients, which may be used as a supplement to the VHI.

## Methods

### Study design

The study was performed in two phases. During phase 1, the original VHI was translated, the Throat subscale constructed and added to the present Swedish version of the VHI. Further, the combined Voice Handicap Index-Throat (VHI-T) was tested for validity and reliability. In phase 2, the VHI-T was re-validated and retested in a large patient-control material.

### Phase 1: Translation of the VHI, development of the Throat subscale. Validity and reliability testing of the VHI-T, experts and responders

An informal, diagnostic instrument with questions on throat related problems has been in use at the phoniatric department since the early nineties. Following the decision to construct an instrument that could be combined with the VHI, the ten symptoms were chosen that had been the most frequently reported during the period of use of the informal instrument. These were suggested as a subscale. The choice of the statements was made in consensus by a panel of experienced phoniatricians and speech therapists. In congruence with the VHI, the items were phrased as statements (Table 1). The statements on throat related symptoms where further commented on and changes suggested by both a panel of experienced clinicians and by patients as described below.

**Table 1:** The statements of the throat subscale with corrected item-total correlation.

Statement	Corrected item-total correlation
1 Jag är torr i halsen (*My throat is dry)*	0.457
2 Jag måste harkla mig (*I need to clear my throat)*	0.625
3 Jag har mycket slem i halsen (*I have a lot of phlegm in my throat)*	0.583
4 Jag känner att det sitter något i halsen (*It feels as if something is stuck in my throat)*	0.683
5 Det svider i halsen (*My throat is burning)*	0.572
6 Jag känner ett tryck utanpå halsen (*I feel a pressure on the outside of my throat)*	0.403
7 Det känns som om jag har en klump i halsen (*It feels like a lump in my throat)*	0.675
8 Jag är irriterad i halsen (*I have an irritation in my throat)*	0.765
9 Jag har ont i halsen (*I have a sore throat)*	0.480
10 Jag har rethosta (*I have a dry cough)*	0.420

Statements are in Swedish, English within brackets

The VHI covers three different domains of voice problems (physical, functional, emotional) and consists of thirty statements, ten in each domain. The statements are phrased in the way the patients normally would express themselves. The occurrence of symptoms are estimated on a frequency-based scale (0 = Never, 1 = Almost Never, 2 = Sometimes, 3 = Almost Always, 4 = Always). In the original questionnaire by Jacobson et al [3], the statements are mixed. In the layout by Rosen and Murry [16], the statements are grouped into three separate domains (commonly called sub scales) with ten statements each. This layout is, in our opinion, more convenient in clinical work. For this translation and adaptation of the VHI into Swedish, the layout proposed by Rosen and Murry was used [16].

When translating an instrument, it is important not only to perform a correct translation but also to make a cross-cultural adaptation of the instrument [17,18]. The original three subscales of the VHI were translated into Swedish by a multidisciplinary experienced expert group of two speech pathologists and three phoniatricians. All items were discussed and language adjustments were made to meet the wordings normally used by our patients.

In purpose to estimate the severity of the self perceived voice problems a 10 cm Visual Analogue Scale was added to the questionnaire, where 0 = no voice problems and 10 = maximal voice problems. This parameter has been used in former studies for estimating the reliability of the VHI [3,19]. The VHI, along with the Throat subscale, were then translated and retranslated to and from English by a professional translator. After a final agreement of the expert group, the questionnaire was submitted to an external group of experts, three phoniatricians, six speech therapists and one singing teacher for comments on the *usefulness *and *face validity *of the questionnaire. Simultaneously, a group of ten consecutive patients were interviewed for comments on the accessibility, the degree of user-friendliness and comments on possible changes to the statements. Further, 150 consecutive patients, referred to the department for voice problems, completed the questionnaire and commented on it. Adjustments of the Throat subscale were done accordingly. For example "I have a sensation of mucus trickling down my throat" was omitted due to rarely being graded higher than 0 or 1. The patients' demographic characteristics or diagnoses were not considered during this phase.

The VHI and the Throat subscale were then submitted to the first phase of testing after minor adjustments. This test-retest procedure included another 40 consecutive patients with voice problems (20 patients with phonastenia and 20 with benign lesions of the vocal folds) and 20 voice-healthy controls from the orthopaedic out-ward department. All responders were to complete two questionnaires with at most one week in between. The first questionnaire was to be completed before the clinical examination and the second to be returned one week later. The two questionnaires were completed and returned in due time by 23 patients (16 F:7 M, median age 54 yrs, range:25-71) and 12 controls (5F:7 M, median age 39, range: 21-71). The testing revealed good reliability. However some items needed rephrasing. Examples of changes: the item "my voice causes me to lose income" (functional scale) that was changed to "my voice restricts my work-life" due to cultural differences between reimbursement systems; "my voice sounds creaky and dry" (physical scale) which was considered by patients to be difficult to answer and was changed to "my voice sounds hoarse" following the phrasing normally used by patients. Therefore the final version of the questionnaire had to be tried once more for both reliability and validity (phase 2).

### Phase 2: VHI-T, revalidation and retesting

This study is based on VHI-Throat questionnaires, i.e. the three original VHI subscales (physical, functional and emotional) along with the Throat subscale. Each maximum subscale-score is 40 p and the total VHI-T is 160 p. The questionnaires were collected from 262 persons. The responders were assigned to four patient groups and one group of controls. To be included, the responders had to be older than twelve years and competent to fill out the questionnaire without help. Twelve/156 patients were excluded due to no response or late return of the second questionnaire. Of the controls, 48/106 persons were excluded due to incomplete questionnaire or late, or no, return of the second questionnaire. This paper thus reports data from 144 patients and 58 controls.

The evaluation of the patients was performed at the Department of phoniactrics, ENT clinic, Lund University Hospital, by the same three phoniatricians with long-lasting, close clinical co-operation, and consensus as to diagnostic criteria of voice disorders. The diagnoses were classified according ICD-10, Swedish version. (Svensk foniatrisk-logopedisk diagnosklassifikation, approved by the Swedish national board of health and welfare 01012000), based on clinical history, videolaryngostroboscopy or high speed filming, and perceptual voice analysis.

The patients were diagnosed with one of the following: *phonastenia *(n = 20;defined by vocal fatigue as a cardinal symptom, without any pathological laryngeal findings, with or without subjective hoarseness); *benign lesions of the vocal folds *(n = 41; 17 polyps; 6 cysts; 5 of each nodules and sulcus glottidis; 3 papillomas; two of each vascular dilatation in the mucosa or atrophy of the vocal folds; and one granuloma); *neurological laryngeal motility disorder *(n = 20; 18 cases with unilateral paresis of the vocal folds and two cases with spasmodic dysphonia); *benign goitre *(N = 41; all referred to the clinic for pre-surgery control), and patients referred for *throat problems *as cardinal symptoms (N = 22), not themselves complaining of voice problems. The Control group (N = 58) consisted of out-ward patients from the orthopaedic department, all reporting voice health and no former contact with voice clinicians. Table 2 presents demographic data on the included responders according diagnose.

**Table 2:** Demographic data for the five groups of patients and one group of voice healthy controls

	Phonastenia	Benign lesions	Neurolog. disorders	**Throat rel**.	Benigngoitre	Controls
**N**	20	41	20	22	41	58
**F:M**	15:5	30:11	12:8	11:11	30:11	31:27
**Median Age (range)**	52 (18-69)	45 (13-74)	56 (26-76)	58 (20-73)	48 (19-79)	60,5 (15-80)

The patients diagnosed with benign goitre and throat related problems were only included for the estimation of the validity. Retesting was not performed in these two groups. The reason for excluding the retesting of the benign goiter group was that the patients were to undergo thyreoid surgery, close after the consultation. The clinical experience is that this surgery may cause slight voice and throat complaints. The patient group with throat problems was included later in the study for the testing of validity and thus did not take part in the retesting procedure.

The reliability of the VHI-Throat was evaluated by a test-retest procedure. The distribution and collection of the questionnaires were identical to the procedure used in phase 1. The questionnaire was first administered to all patients on arriving for their primary consultation at the phoniatric department, to be completed before the clinical examination. After one week, a new questionnaire was sent to all the patients, to be completed and returned within one more week. The Controls completed the questionnaire at the orthopedic out-ward department. They were given the second questionnaire at the same occasion, and were asked to return it within two weeks. The reason for using a different way of distributing the second questionnaire to the controls, was based on earlier experience from phase one. Namely, the control persons did not return the second questionnaire when it was mailed to them. The compliance improved when the second questionnaire was handed to the controls after the completion of the first. The validity of the VHI-Throat was assessed by comparing the whole group of patients to the group of controls.

### Statistics

The test-retest reliability for the VHI- Lund total scores, for the values of the subjective voice estimation, and for the Throat subscale was estimated by calculating the IntraClass Correlation coefficient (ICC). For the construct validity, independent samples t-tests were used to compare the average scores of the VHI-Throat total, subjective voice estimation values and the Throat subscale between patients and controls. The Pearson product-moment correlation coefficient was used for computing the correlations between the subscales and the VHI-Throat total score, the throat subscale and the original VHI subscales and for estimating the correlation between the subjective assessment of voice and VHI-Throat total score. The internal consistency and reliability of the total VHI-Throat subscale, as well as of the throat subscale, were calculated with inter-item correlation and Cronbach's alpha coefficient. An ANOVA was performed to further analyze the VHI-T subscales. Analyses were performed using SPSS 15.0 and 16.0 for Windows. Alpha levels were set at 0,05%. (, ICC) and 0,01% (Pearson)

### Ethical aspects

The study was approved by the ethical committee at Lund University (No LU 366-01).

## Results

### The throat subscale, validation process

The statements of the throat subscale are presented in Table 2. The *face *and *content validity *were tested during phase 1, see Methods section above. The *test-retest reliability *of the throat subscale was estimated with ICC: *r *= 0,871, in 144 patients and 58 controls, proving the scale to be stable and reliable.

#### Construct validity and internal consistency

The average score of the throat subscale in all 144 patients (M = 13,5. Sd = 6,8) was significantly different from that in the controls (M = 6,9 Sd = 5,5), t(178) = 6,8, *p *< 0.01, proving the throat subscale to be sensitive enough to differentiate between subjects with throat problems and healthy controls (Table 3). The Cronbach's alpha coefficient for the throat subscale was *r *= 0,87. In Table 2, all statements of the throat sub scale are given along with the corrected item-total correlations, reflecting the degree to which each statement correlates to the total score of this scale. The criterion for inclusion of an item in a subscale is an item-total correlation of > 0.3. As shown in Table 1, the corrected item-total correlations for all statements exceeded 0.4, thus indicating satisfactory correlation of the statements within this subscale. When item-total correlation was calculated for all items of the VHI-T (Appendix), the values were somewhat lower for the **t**hroat subscale, however no item scored < 0.3.

**Table 3:** Results of T-test between patients and voice healthy controls for the VHI-Throat subscales.

		M score (Sd)	t	df	P = (2-tailed)
**Throat scale**	Patients	14,5 (7,3)			
	Controls	6,9 (5,5)	8,1	138	,001
**Functional**	Patients	9,5 (9,7)			
	Controls	1,8 (3,4)	8,3	197	,001
**Physical**	Patients	15,1 (9,8)			
	Controls	5,4 (5,6)	8,8	178	,001
**Emotional**	Patients	8,7 (9,5)			
	Controls	1,3 (3,1)	8,4	194	,001

Patients n = 144, Controls n = 58

### VHI-Throat: the VHI questionnaire and the throat subscale, reliability and validity

#### Test-retest reliability, construct validity and internal consistency

The test-retest reliability of the total VHI-T score was estimated with IntraClass coefficient (ICC): = 0,968, proving good reliability of the questionnaire. A paired samples revealed no significant differences between the first and second occasion for neither the total VHI-T scores (M = 1,6, Sd = 41,6, N = 142), *t*(141) = 0,464, *p *= 0,6 nor the individual subscale scores (Throat: (M = 0,9, Sd = 10,4, N = 142), *t*(141) = 1,0, *p *= 0,2, Functional: (M = 0,5, Sd = 12,4, N = 142), *t*(141) = 0,526, *p *= 0,6, Physical: (M = 0,3, Sd = 13,1, N = 142), *t*(141) = 0,351, *p *= 0,7, Emotional: (M = -0,3, Sd = 13,2, N = 142), *t*(141) = -0,2, *p *= 0,8) in patients and controls. The VHI-T total score in all patients (M = 47,8, Sd = 30,2, N = 144) was significantly higher than in the controls (M = 15,3SD = 15,0N = 58), *t*(191) = 10,2, *p *< 0.05 (2-tailed), thus indicating that the questionnaire separated persons with and without voice pathology. Independent Samples t-tests were also calculated for the subscales, showing significant differences between patients and controls for the three original subscales and the throat subscale. (Table 3) The Cronbach's alpha coefficient was *r *= 0,90 for the total VHI-T scale and *r *= 0,93 if the throat subscale would be excluded. There was a strong correlation between each of the four subscales and the total score for VHI-T, respectively, as shown by Pearson's correlation coefficient: throat subscale *r *= 0,684, functional scale *r *= 0,921, physical scale *r *= 0,931 and emotional scale *r *= 0,915. A one-way analysis of variance showed significant differences at the p < .05 level in subscale scores between the groups of patients: Throat scale: F(5,193) = 18,4, *p *= .000; Functional scale: F(5,193) = 48,1, *p *= .000; Physical scale: F(5,193) = 57,7, *p *= .000; Emotional scale: F(5,193) = 37,4, *p *= .000. Further analysis with Tukey HSD test for the Throat scale indicated statistically significant differences between the mean scores for the phonastenia group (*M *= 14,8, *Sd *= 6,3) and the control group (*M *= 6,9, *Sd *= 5,7); between the benign lesions group (*M *= 15,8, *Sd *= 6,7) and the benign goiter group (*M *= 10,3, *Sd *= 6,4) as well as the control group (*M *= 6,9, *Sd *= 5,7); between the benign goiter group (*M *= 10,3, *Sd *= 6,4) and throat related group (*M *= 19,8, *Sd *= 5,6); between the neurolog. disorder group (*M *= 14,1, *Sd *= 8,1) and the throat related group (*M *= 19,8, *Sd *= 5,6) as well as the control group (*M *= 6,9, *Sd *= 5,7).

### The relation of the throat scale and the VHI

The correlation between the throat scale and the three original VHI subscales was calculated with Pearson's correlation coefficient: functional scale: *r *= 0,356 physical scale: *r *= 0,544; emotional scale: r = 0,395, thus suggesting a moderate to strong correlation with the physical scale and a moderate correlation with the functional and emotional subscales.

### The relation of the throat scale to the VHI-T total score

The mean scores of the four VHI-T subscales and the VHI-T total score for each diagnose group are presented in Table 4. Table 4 also shows the relation between each subscale and the total scores of the VHI-T in percent and thus indicates the dominating subscale or subscales for each diagnose. The diagnoses follow two different patterns based on the relation between the subscale-scores. The distribution of the scores for the neurological disorders, benign lesions and phonastenia is even, with close to 25% for each subscale. The throat subscale scores for benign goiter and throat-related disorders account for more than 50% of the total VHI-T score.

**Table 4:** Mean scores of the VHI-T subscales, percentage of the subscales of the total VHI-T scores.

	Throat		Functional		Physical		Emotional		Tot VHI-T	
	M (Sd)	*%*	M (Sd)	*%*	M (Sd)	*%*	M (Sd)	*%*	M (Sd)	*%*
**Neurological **N = 20	14 (8)	*20*	19 (8)	*27*	21 (6)	*30*	16 (8)	*24*	70 (22)	*100*
**Ben. Lesions **N = 41	16 (7)	*23*	16 (9)	*22*	29 (7)	*42*	15 (10)	*22*	70 (27)	*100*
**Phonastenia **N = 20	15 (6)	*30*	10 (7)	*20*	16 (6)	*34*	9 (6)	*18*	49 (19)	*100*
**Ben. Goitre **N = 41	10 (6)	*52*	2 (5)	*12*	6 (6)	*29*	1 (4)	*8*	20 (18)	*100*
**Throat rel **N = 22	20 (7)	*56*	2 (2)	*5*	10 (7)	*28*	4 (5)	*11*	36 (15)	*100*
**Controls **N = 58	7 (5)	*45*	2 (3)	*12*	5 (6)	*35*	1 (3)	*9*	15 (15)	*100*

### Subjective estimation of the voice with Visual Analogue Scale (VAS)

#### Test-retest reliability and construct validity

The reliability of the subjective estimation of the voice was calculated by ICC and showed a moderate-strong correlation: *r *= 0,712, N = 202, *p *< 0,05, proving it as a satisfactory stable instrument. Calculation with independent T-test showed that the difference in the subjective estimation of the own voice between the patient-group (M = 43,8 Sd = 31,2 N = 122) and control-group (M = 14,3 Sd = 19,8 N = 58), was significant *t(*163) = 7,7, *p *< 0,05. The results indicate that this instrument was sensitive enough to separate patients from controls.

#### Correlation between estimation of one's own voice and VHI-T total score

The correlation between the subjective estimation of the voice and the total VHI-T was a moderate when tested in all patients and controls using Pearson's product-moment correlation coefficient (*r *= 0.79 n = 202, *p *< 0.01.) For the different groups the correlation coefficient varied: Phonastenia group (0,48), Benign lesions (0,69), Neurological group (0,70), Benign Goitre (0,68), Throat related disorders (0,64), and Controls (0,32).

## Discussion

### The need to estimate throat problems in the voice clinic

In the Swedish healthcare system, patients with a broad spectrum of voice and voice related problems are diagnosed and treated at logopedic-phoniatric departments. In our daily practice, we have experienced that many patients report more physical aspects than those covered by the original VHI domains (functional, physical, and emotional domains). This was the impetus to create the throat subscale. Throat problems are ascribed to a multitude of etiologies, are common in voice patients and considered to be cardinal symptoms in patients with vocal fatigue. The need of a structured broader aiming instrument, for the self-assessment of the problems patients report in the voice clinic has also been emphasized by Deary et al [8] and Glas et al [20]. We share the view of these authors that the spectrum of patient-reported problems in the voice clinic is broader than the "classical" voice symptoms, and are not uncommonly symptoms that originate from throat.

### VHI-Throat, a questionnaire

The VHI-Throat (VHI-T) questionnaire showed good test-retest reliability, validity and internal consistency. According to the present results, it seems that the throat subscale fends for itself as indicated by the Cronbach's alpha value as well as the corrected inter-item correlation analysis (see Table 1 and additional file 1) and by the correlation between the throat scale and the original three VHI subscales. The total score and the scores of the three original VHI subscales were comparable to those in corresponding groups of patients in other studies [4,19,21]. The VHI-T thus seems to be an appropriate tool for clinical use in Swedish speaking populations, also being patient-friendly and convenient to administer and evaluate.

Our results show that the Throat-subscale in combination with the VHI is an instrument that may make it possible to discriminate between voice and throat problems and to help the patient express both categories of concerns simultaneously. To our knowledge, until today there has been no instrument developed for the estimation of the patient's overall description of symptoms in the voice clinic, where many patients with throat-problems are referred. A deeper insight in the problems may lead to an increased understanding of the patient with throat complaints, with or without voice complaints. This knowledge may be helpful in designing the clinical intervention. However, it does not give us any indication of the origin of the problems.

Our results from the voice-healthy subjects show that it is not uncommon to report some symptoms from the throat. Moreover, our results indicate that patients who report problems mainly from the throat also have some complaints on the physical subscale. This is in accordance with the findings of Belafsky et al, who found a decrease on the physical subscale after the treatment of laryngeal reflux [14]. We believe that the VHI-T may become a useful clinical instrument that may help to discriminate the problems that might be either co-existing or occurring separately. However, sharing the opinion of Verdonck et al [21], to be able to pin-point the focus of the patient's problems it might be more rewarding to evaluate the subscale scores of the VHI, rather than the total score.

The way of collecting the second questionnaire (see methods) might of course have brought bias into the results. Based on earlier experience, the second questionnaire was given to the voice-healthy controls already at the completion of the first questionnaire, where the patients were sent the second questionnaire by mail. Even though all subjects included returned the second questionnaire within two weeks, we have no means of knowing when the second questionnaire actually was completed by the controls.

### The VHI and the VHI-Throat

The Voice Handicap Index is today widely used in clinic and research. Despite some recent critical opinions that the VHI lacks statistically discrete subscales [4], it still fills the purpose of covering the self perceived voice problems and also the consequences for the quality of life that voice disorders may lead to. We have used a Swedish translation of the VHI in clinic since 2000 and it was therefore natural to choose the VHI as a base for the development of the throat subscale.

The use of VHI and other self-reporting instruments within the voice clinic has had an eye-opening effect since the patient's own estimation of the symptoms thus has come more into focus. The VHI-T is designed as an instrument for the patient to estimate the perceived problems and, in our experience the throat subscale is a good complementary tool to the VHI, allowing a better identification of actual disorders. Consequently, we can better design more appropriate therapeutic interventions. Some patients call for medical consultation specifically due to throat-related symptoms, but quite often the referring physician may interpret the symptoms as signs of a voice disorder. The use of the compiled VHI-T may thus direct the clinician to a more appropriate intervention.

Interestingly, our results indicate that it may be possible to identify two "profiles" of symptoms characterising different groups of patients. As is evident in Table 4, voice healthy controls-, benign goitre- and throat-groups report the lowest total VHI-T scores (15-36) but the percentage of their indicated throat problems is high relative to the total score. Conversely, the patients with benign laryngeal lesions report the highest VHI-T total score (70) with rather equal distribution of symptoms over the four subscales. Further studies are, however, necessary in order to estimate the usefulness of "profiles" for the clinical evaluation of individual patients. The ANOVA showed significant differences in the subscale scores between the patient groups. However, we wish to be cautious in interpretation of these findings. The VHI is a self rating instrument of symptoms and has as such not been intended as a differential diagnostic instrument. The differences between the patients' "profiles" emerging from Table 4, may however, be used for evaluating the effect of therapy within individual patients. Since the results of the validation of the original VHI-subscales within this study are in accordance with the results of other studies [3,21,22] we may suggest that the throat subscale can be used for clinical and research purposes along with any validated VHI version.

### The subjective estimation of the voice with VAS

The subjective estimations of the voice with VAS showed good test-retest reliability. Correlations between the subjective estimations of the voice and the overall VHI-T score were reliable in the whole population but varied between the different diagnostic groups. Subjective estimation of voice is usually used only for proving the face validity of the VHI-questionnaire [3,5,19]. We choose to include this simple measure as a permanent item in the questionnaire. It gives a quick overview of the patient's own grading of the voice problems [1].

As in other studies [3,5,19], we also found a good correlation between the average scores from the subjective estimation of the voice and the total score of VHI-T, however with varying correlations between the diagnose groups. A discrepancy between VHI-T and VAS may be of interest since it may reflect the patient's attitude to his/her symptoms: a patient who has a combination of high VHI-T total score and a low value of self-estimation of the voice may in fact not value the symptoms as a big trouble while another individual with the reverse relationship between the self-estimation of the voice and VHI-T total values the symptoms as less tolerable. This information cannot be underestimated when taking care of the patients in voice therapy, not least since it may actually give a hint of the patient's motivation to complete the therapy.

## Conclusions

The present Swedish translation of the VHI with the subscale on throat-related problems, the VHI-Throat, proves to be a valid and reliable instrument for the estimation of self-perceived voice and throat problems. The use of the throat subscale helps to reveal a category of symptoms that are common in our patients and that are only marginally covered in other available instruments. In analogy with other translations of the VHI, it can be used for both clinical purposes and for clinical research.

## Competing interests

The authors declare that they have no competing interests.

## Authors' contributions

This work is performed in close collaboration by the authors. VLÅ and LS initiated, designed and coordinated the study and also carried out the distribution of the questionnaires. VLÅ analysed the data and drafted the manuscript. LS helped discussing and drafting the manuscript and examined the patients. JE collected and analysed the data for the voice- healthy controls under supervision of LS and VLÅ. RR examined the patients, participated in the expert group and helped drafting the manuscript. All authors read and approved the final manuscript.

## Authors' informations

VLÅ: Speech therapist MSc. PhD student at the dep of logopedics, phoniatrics and audiology, Lund University. LS: MD, PhD, consultant in phoniatrics, ENT dep Lund University Hospital. RR: MD, PhD, consultant in phoniatrics and laryngology, ENT dep Lund University Hospital and dep of logopedics, phoniatrics and audiology, Lund University. JE: Speech therapist MSc, dep of logopedics, phoniatrics and audiology, Lund University.

## Pre-publication history

The pre-publication history for this paper can be accessed here:

http://www.biomedcentral.com/1472-6815/10/5/prepub

## Supplementary Material

Additional file 1 **Values of the corrected item-total correlation between the statements of the VHI-T**. This file represents a table showing all statements in the VHI-T in Swedish and English, and also showing the values of the corrected item-correlation between the statements. Click here for file
