# Supplementary material for: Throat related symptoms and voice: development of an instrument for self assessment of throat-problems
Source: BMC Ear Nose Throat Disord. 2010 May 27;10:5. doi: 10.1186/1472-6815-10-5 (PMC2891628; doi:10.1186/1472-6815-10-5)
Supplement: Additional file 1 — Values of the corrected item-total correlation between the statements of the VHI-T. This file represents a table showing all statements in the VHI-T in Swedish and English, and also showing the values of the corrected item-correlation between the statements. [file 1472-6815-10-5-S1.DOCX]

**Additional files**

***Additional file 1***

***Title:*** *Values of the corrected item-total correlation between the statements of the VHI-T*

***Description:*** *This file represents a table showing all statements in the VHI-T in Swedish and English, and also showing the values of the corrected item-correlation between the statements.*

| **Statement** | **Corrected Item-Total Correlation** |
| --- | --- |
| T1 Jag är torr i halsen (*My throat is dry.)* | ,408 |
| T2 Jag måste harkla mig (*I need to clear my throat.)* | ,539 |
| T3 Jag har mycket slem i halsen (*I have a lot of phlegm in my throat.)* | ,449 |
| T4 Jag känner att det sitter något i halsen (*It feels as if something is stuck in my throat.)* | ,498 |
| T5 Det svider i halsen (*My throat is burning.)* | ,478 |
| T6 Jag känner ett tryck utanpå halsen (*I feel a pressure on the outside of my throat.)* | ,464 |
| T7 Det känns som om jag har en klump i halsen (*It feels like a lump in my throat.)* | ,512 |
| T8 Jag är irriterad i halsen (*I have an irritation in my throat.)* | ,662 |
| T9 Jag har ont i halsen (*I have a sore throat.)* | ,434 |
| T10 Jag har rethosta (*I have a dry cough.)* | ,384 |
| F1 Andra har, på grund av min röst, svårt att uppfatta vad jag säger.(*My voice makes it difficult to hear me.*) | ,810 |
| F2 Jag har svårt att göra mig hörd i bullrig miljö, som t ex på ett kalas. *(people have difficulty understanding me in a noisy room.)* | ,804 |
| F3 Jag har svårt att öka röststyrkan för att ropa. *(My family has difficulty hearing med when I call them throughout the house.)* | ,830 |
| F4 Jag undviker att tala i telefon på grund av min röst. *(I use the phone less often than I would like to.)* | ,658 |
| F5 Människor ber mig upprepa vad jag har sagt. *(People ask me to repeat myself when speaking face-to-face.)* | ,742 |
| F6 Jag undviker att tala i grupp på grund av min röst. *(I tend to avoid groups of people because of my voice.)* | ,737 |
| F7 Jag talar mer sällan än jag skulle vilja med vänner och familj på grund av min röst. *(I speak with friends, neighbors, or relatives less often because of my voice.)* | ,687 |
| F8 Min röst begränsar mig i mina fritidsaktiviteter. *(My voice difficulties restrict personal and social life.)* | ,600 |
| F9 Jag blir utelämnad ur samtal på grund av min röst. *(I feel left out of conversations because of my voice.)* | ,584 |
| F10 Min röst begränsar mig i mitt arbetsliv *(My voice problem causes me to lose income)* | ,738 |
| P1 Luften tar slut när jag talar. *(I run out of air when I talk)* | ,670 |
| P2 Kvaliteten på rösten varierar under dagen. *(The sound of my voice varies throughout the day.)* | ,689 |
| P3 Andra frågar om jag är förkyld. *(People ask, “What’s wrong with your voice?”)* | ,683 |
| P4 Min röst låter hes. *(My voice sounds creaky and dry)* | ,833 |
| P5 Min röst är sämst på morgonen *(I feel as though I have to strain to produce voice.)* | ,375 |
| P6 Min röst kan plötsligt förändras under ett kortare samtal. *(The clarity of my voice is unpredictable.)* | ,776 |
| P7 Jag försöker förändra min röst för att låta bra. *(I try to change my voice to sound different.)* | ,642 |
| P8 Det är ansträngande att tala. *(I use a great deal of effort to speak)* | ,828 |
| P9 Min röst är sämst på kvällen. *(My voice is worse in the evening)* | ,653 |
| P10 Rösten försvinner mitt i en mening. *(My voice “gives out” on me in the middle of speaking.)* | ,729 |
| E1 På grund av min röst spänner jag mig när jag talar med andra. *(I am tense when talking to others because of my voice.)* | ,802 |
| E2 Andra verkar bli irriterade på min röst. *(People seem irritated with my voice.)* | ,710 |
| E3 Andra verkar sakna förståelse för mina röstproblem. *(I feel other people don’t understand my voice problem.)* | ,697 |
| E4 Mina röstproblem gör mig orolig. *(My voice problem upsets me.)* | ,796 |
| E5 Jag är mindre utåtriktad på grund av mina röstproblem. *(I am less outgoing because of my voice problem.)* | ,766 |
| E6 Jag känner mig handikappad på grund av min röst. *(My voice makes me feel handicapped.)* | ,830 |
| E7 Jag blir irriterad när andra ber mig upprepa vad jag sagt. *(I feel annoyed when people ask me to repeat.)* | ,667 |
| E8 Jag känner mig besvärad när andra ber mig upprepa vad jag sagt. *(I feel embarrassed when people ask me to repeat.)* | ,703 |
| E9 Min röst gör att jag känner mig osäker. *(My voice makes me feel incompetent.)* | ,747 |
| E10 Jag skäms för mina röstproblem. *(I am ashamed of my voice problem.)* | ,666 |
